# Land-Use Planning Serves as a Critical Tool for Improving Resources and Environmental Carrying Capacity: A Review of Evaluation Methods and Application

**DOI:** 10.3390/ijerph20032370

**Published:** 2023-01-29

**Authors:** An Huang, Li Tian, Qing Li, Yongfu Li, Jianghao Yu, Yuan Gao, Jing Xia

**Affiliations:** 1 School of Public Administration, Xi’an University of Architecture and Technology, Xi’an 710055, China; 2 School of Architecture, Tsinghua University, Beijing 100084, China; 3 Innovation Center for Smart Human Settlements and Spatial Planning & Governance, Ministry of Natural Resources, Beijing 100084, China; 4 College of Architecture and Urban Planning, Tongji University, Shanghai 200092, China; 5 Shanghai Academy of Fine Arts, Shanghai University, Shanghai 200444, China; 6 School of Landscape Architecture, Beijing Forestry University, Beijing 100083, China; 7 College of Architecture & Art, Hefei University of Technology, Hefei 230009, China

**Keywords:** resources and environmental carrying capacity (RECC), RECC assessment methods, land-use planning, literature review

## Abstract

Research on resources and environmental carrying capacity (RECC) has been expanding since the early 20th century, and RECC has become a global concern and criterion for measuring regional sustainable development. Land-use planning (LUP) serves as a key tool of socioeconomic–ecological coordinated development and is deeply associated with RECC. In China, the newly established spatial planning system of 2019 identifies RECC assessment as the basis for spatial planning. However, after systematically reviewing the research history, conceptual evolution, and evaluation methods of RECC, we found that the existing approach of RECC has not addressed the impacts of stakeholders’ behavior on RECC, in other words, the governance perspective has not been sufficiently discussed. Further, research on the interaction between RECC and LUP has been far from sufficient, hampering our deep understanding of the roles of LUP in improving RECC. In order to fill this gap, a new framework is proposed to explain the formation mechanism of RECC combining the governance considerations based on the social–ecological system (SES) framework, which has made contributions to enrich the research perspective of RECC and its theoretical and methods system. In addition, the interaction path between RECC and LUP is constructed according to the new RECC framework and a policy toolbox for improving RECC, which will provide a comprehensive and systematic practical application path for improving RECC and promoting regional socioeconomic–ecological coordinated sustainable development. The conclusion part discusses the future research topics and limitations for RECC and LUP.

## 1. Introduction

Land-use planning (LUP) is a key tool to guide the spatial layout and time sequence of land use and to minimize the negative externality of land use. Resource and environmental carrying capacity (RECC) is an important yardstick for guiding the practice of LUP towards sustainable regional and urban development. RECC measures the interaction between human activities and the urban resources environment system, and identifies key constraints affecting their coordinated development. It has been widely used in the research on the carrying capacity of water resources, water environment, land resources, and atmosphere [1,2,3,4]. As early as the beginning of the 20th century, Pfaundler [5] and the USDA Yearbook [6] put forward the concept of RECC. Since the end of the 20th century, given the problems of resource depletion and environmental degradation, RECC has been gradually and widely applied in regional ecosystem services assessment, population planning [7], economic development planning [8], land planning [9], water resources and environmental planning [10], ecological and economic planning [11,12], resource and environment planning and management [13,14,15], and global and regional sustainable development planning [16,17,18]. The research and application scope can be considered as the thematic planning of land use planning. Meanwhile, the Future Earth program—an important strategic research agenda—was proposed in 2012 to integrate research on RECC with global change and socioeconomic sustainable development [19]. Since the beginning of the 21st century, RECC has been introduced in China’s major-function-oriented zoning planning to describe development constraints [20], in post-disaster reconstruction planning [21], in territorial spatial planning [22,23], and in setting goals for social and economic development [22,24]. Those research projects show that LUP serves as a key tool of socioeconomic–ecological coordinated development and is deeply associated with RECC. However, a clear understanding of the interaction pathway between RECC and LUP has not been formed, for instance, how to evaluate the impact of LUP on RECC? What tools in LUP can be applied to improve RECC, and what are the improvement paths? These critical issues require further in-depth research.

This paper is organized as follows: It begins with a literature review on the evolution of the RECC concept. The next section constructs a RECC framework by combining the social–ecological system (SES). Section 3 reviews the RECC assessment and calculation methods. Then, the interaction pathways between RECC and LUP are summarized. The last section describes the outlook for RECC and its future applications in LUP.

## 2. Evolution of the RECC Concept

The concept of carrying capacity comes from mechanics and refers to the maximum load that an object can carry without any damage. Carrying capacity can be measured experimentally or empirically. Subsequently, carrying capacity has been cited in, expanded on, and improved by the fields of ecology, resource science, and environmental science, to form the present concept of RECC. The stages of the RECC concept development can be divided as follows.

*(1) The emergence of the RECC concept (1798–1940)*. The introduction of carrying capacity into the field of resources and environment can be traced back to Malthus’ An Essay on the Principle of Population [25]. His theory holds that population increases at a geometric rate, and means of living increases at an arithmetic rate. Population growth tends to exceed means of living, and there are positive controls (hunger, war, poverty) and preventive controls to curb population growth. In this way, the concept of population carrying capacity has been endowed with a modern connotation. Park [26] extended the concept of carrying capacity into the field of human ecology, believing that carrying capacity is the limit on the number of individual organisms under a specific environmental condition (mainly referring to the combination of living space, nutrients, sunlight, and other ecological factors). Hadwen [27] further clarified the interaction between animal population and the environmental state, proposed the concept of grassland carrying capacity from the grassland ecological perspective, and extended the focus from the initial population capacity limit to the balance of the eco-environment. Based on ecological perspective, Leopold [28] proposed biomass carrying capacity, which is the capacity of an ecosystem to carry the maximum amount of a particular biomass at a time. His research raised awareness of limited resources and environmental development, and laid a foundation for the later carrying capacity research and sustainable development theory.

*(2) The development stage of the RECC concept (1940–1990)*. The concept of carrying capacity evolved from the concern of natural ecosystem to the concern of the relationship between humans, resources, and the environment, which is the transition point of stage development [29]. The study of carrying capacity flourished after the end of World War II. To deal with disasters like hunger, war, and poverty, Aldo Leopold [30] put forward the concept of a human carrying capacity in 1943, referring to how many people can be held per unit area, which gradually evolved into the population number that can be carried or supported by natural resources per unit area or region in later studies. With the advance of global industrialization, large-scale urbanization has reduced the amount of arable land and strained food supplies, posing new challenges to the ability of land resources to supply food. Thus, based on the study of agriculture and animal husbandry in Africa, Allan [31] proposed land carrying capacity in 1949. Meanwhile, the threat of industrial pollution damage to the ecological environment became increasingly prominent. Researchers gradually realized the contradictory and interdependent relationships between ecosystems and people. For example, The Limits to Growth [8] is an outstanding in-depth discussion of the global population carrying capacity and related resource and environmental issues. 

Over time, research objects became increasingly complex. The conceptual core changed from phenomenon description to mechanism analysis, and the concept changed from static balance to dynamic change, and further, to the field of sustainable development [20]. For instance, the United Nations Educational, Scientific, and Cultural Organization [32] defined resource carrying capacity as “the number of population that a country or region can sustainably support within a foreseeable period under the material living standard conforming to its social and cultural standards by using local energy and other natural resources and intellectual and technological conditions.” The Food and Agriculture Organization [33] carried out research on the potential population carrying capacity of land in developing countries, and applied the concept of population, resources, and environment interaction in the making of development planning. The purpose of the research extended from population balance to social decision making, and the nature of carrying changed from the absolute upper limit to a relative balance.

Since the 1970s, the world has experienced the most serious environmental damage and pollution since the Industrial Revolution, such as the most typical case of the pollution of the Thames caused by the population surge in Britain from 1849 to 1960 [34]. Environmental carrying capacity was therefore proposed by Portmann [35] in 1986, which refers to an attribute of the environment defined as the ability to accommodate specific activities without causing unacceptable impacts. In the later research and application, environmental carrying capacity is gradually expanded and extended, forming a complete concept including water, atmosphere, and soil environment [36].

*(3) The wide application and comprehensive stage (1990–)*. With global warming, resource depletion, environmental deterioration, food crises, and ecological crises constantly exposed and highlighted, sustainable development has garnered enormous attention. Further, global sustainable development reached consensus in the Rio Declaration and Earth Summit: Agenda 21 adopted by the United Nations Conference on Environment and Development in 1992, which marked the beginning of this stage. The concept of global sustainable development promotes carrying capacity from concept, theory, and scientific research to management practice. Carrying capacity thus becomes an essential quantitative tool for sustainable development [29]. In addition, since it is difficult to accurately measure the extreme value or the threshold of carrying population, the path of carrying capacity calculation was once changed to measuring the area of planet Earth required by a specific population size—namely the ecological footprint method [37]—which also indicates that RECC concept research has entered a wide application and comprehensive stage. In this stage, the concept of carrying capacity is gradually put forward and improved in the process of planning and application, such as, the concept of water carrying capacity for water shortage [38], mineral resources carrying capacity for mineral resource shortage [39], ecological carrying capacity for ecological civilization construction [40], resources and environmental carrying capacity for post-disaster reconstruction planning [41] and territorial space planning [42], culture carrying capacity for cultural protection [43], product–living–ecological carrying capacity for product–living–ecological space coordination development [44], resources and environmental carrying capacity of rural and township development for rural revitalization [22,45], economic carrying capacity [24] and traffic carrying capacity [46] for regional and urban sustainable development, and tourism carrying capacity for tourism sustainable development [47].

**Figure 1:**
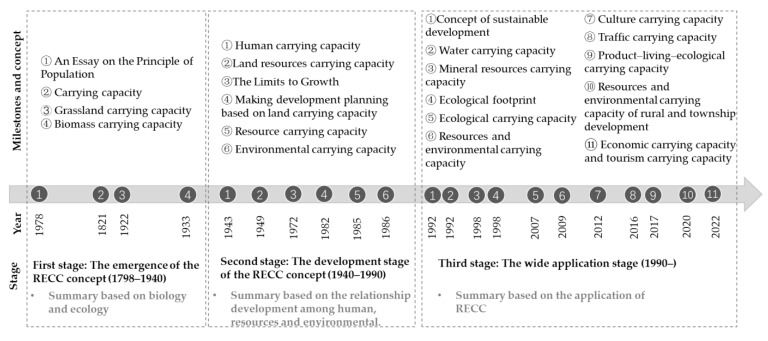
Milestones and concept of study on RECC.

Therefore, since the introduction of the concept, RECC has evolved from a single-factor consideration to the synthetic carrying capacity, from basic concept to applied concept, and its system elements have increased dramatically. Milestones and concept of study on RECC are illustrated in Figure 1 and the evolution of the RECC concept is shown in Table 1.

## 3. The RECC Formation Mechanism

RECC is a complex system with multiple dimensions (e.g., social, ecological, governance), levels, and elements [23]. Many RECC frameworks have been formed through analyzing resource support, environmental and ecological factor constraints, and socioeconomic factor pressures, such as the minimum restrictive factor framework, the multifactor synthesis framework, the pressure–carrying state spatial framework, and the ecological footprint framework [22,36,44]. However, to date, the impact of governance on RECC has not been sufficiently addressed. Although there has been much research on the influence of stakeholder actions on RECC, the research of combining governance and other subsystems, such as socio-economic and environment systems, has been insufficient. 

**Figure 2:**
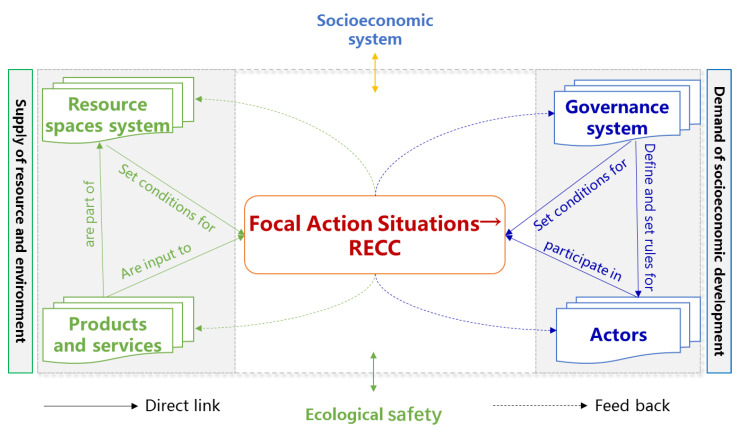
An SES framework of RECC. Note: Adapted from McGinnis et al. [54].

The social–ecological system (SES) framework has been put forward by Ostrom [52]. The SES framework is a socioecological complex system visualization integrated with a multicenter governance system [52,53]. It established an explanatory framework for sustainable development by introducing the interaction pathway among social, ecological, governance, stakeholders, etc. Therefore, it can be applied to fill the gap in the lack of governance consideration in RECC assessment and improvement. This framework identifies and deconstructs exclusive and competitive public resource governance issues, socioecological processes, and their key variable interrelations from multiple dimensions, such as resources system, resource unit, actors, governance system, interaction scenarios, and outcome. The RECC based on the SES framework is shown in Figure 2.

On the premise of ecological safety and socioeconomic sustainability, driving the different demands of socioeconomic development, stakeholders extract resources and environmental products and services from the resources systems (e.g., water, construction land, cultivated land, ecological) according to the relevant rules. The scenarios vary from the different actions of stakeholders, including government, market, and organizations, incurring the change of socioeconomic development and the supplies of resources and the environment. While assessing RECC, we identify the difference between the supply and demand as the RECC state value. When the RECC value is negative, resources and environments are overloaded. When the RECC value is positive, the resources and environments are loadable. When supply and demand are infinitely close to zero, they are infinitely close to a coordinated and balanced state between resources and environments and socioeconomic development. Each subsystem makes dynamic adjustments according to the RECC value feedback to ensure coordinated operation between the social and ecological system. Six subsystems are described as follows: 

*(1) Resources spatial system.* This subsystem is the carrier of the resource and environmental products and services. The spatial scale determines the upper limit of the capacity to supply products and services, while the spatial structure is a key factor affecting the capacity to supply the products and services.

*(2) Products and services.* This subsystem refers to the quantity and quality of specific products that the resource spatial system supplies to socioeconomic development, such as total water resources, water environmental quality, construction land scale, outputs of secondary and tertiary industries, total agricultural production, total grain production, forest land coverage rate, and grassland output.

*(3) Governance system.* This is a set of guidelines for action developed by a government or non-government organization and individuals, and includes government investment and policy intervention, bottom-up collective spontaneous action, foreign investment, and the participation of non-governmental organization and individuals [55]. The impact of stakeholder actions on RECC might be positive or negative. 

*(4) Actors.* This subsystem encompasses the type, number, and structural changes of the actors, which have a great impact on resource and environmental use intensity [53]. For example, the total demand of water and land area may be estimated according to the population and the quotas of water and land area for production, living, and ecology. 

*(5) Socioeconomic system.* Situation and goals in the socioeconomic system is critical for sustainable development. Growth in resident populations and local economies will inevitably lead to increased demand for water, land, and ecological uses, and government policies may exacerbate or mitigate these effects [56].

*(6) Ecological safety.* Ecological safety is a prerequisite for sustainable social–ecological development, which requires the governance system to provide a set of scientific threshold systems to ensure the bottom line of ecological security [22,23].

*(7) Focal Action Situations → RECC.* This subsystem is the core of the framework. According to the goal of coordinated socioeconomic and ecological development, and the carrying results feedback, the focal governance system responds to and adjusts the social and economic demand as well as resources and environmental use to ensure that the RECC is at the appropriate scope. The types of RECC include water resource carrying capacity, water environment carrying capacity, construction land carrying capacity, grain carrying capacity, ecological carrying capacity, etc.

According to the above analysis, the RECC concept can be improved. On the premise of ecological safety and socioeconomic sustainability, the spaces, products, and services of resources and environments can hold capacity for stakeholders. That is true if they follow the governance rules to engage in production and living activities in order to strike the balance between eco-environment and socio-economic development. Moreover, LUP is a key part of the governance system in the SES framework. Thus, the RECC results can be positively or negatively affected by the regulation rules of key factors in different LUP schemes [57,58]. Meanwhile, as the quantitative indicators for sustainable regional development, the RECC results can be used as the basis for decision making in the LUP scheme [2,59] and the earning warming for the implementation of LUP [60,61].

## 4. The RECC Assessment and Measurement Methods

**Figure 3:**
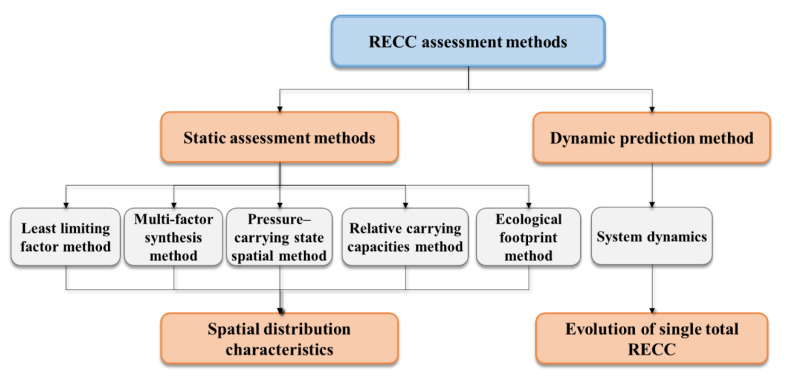
RECC assessment methods.

Figure 3 summarizes the existing RECC assessment and calculation methods. These can be roughly divided into static assessment and measurement methods and the dynamic prediction method. Static assessment methods evaluate the current RECC (dimensionless/dimensional), usually in the form of spatial distribution characteristics. These include the least the limiting factor method, multifactor synthesis method, pressure–carrying state spatial method, opposable mind method, and the ecological footprint method. The dynamic prediction method uses a system dynamics (SD) model to simulate the long-term evolution of a single total RECC value. The formation background, logical starting point, and advantages/disadvantages of these methods are given in what follows.

### 4.1. The Least Limiting Factor Method

Food crises, environmental deterioration, resource shortages, and other crises have brought more attention to RECC. The authors of [62] took food shortage as an example and developed a creative evaluation system based on the least limiting factor, which has been developed and matured in a series of subsequent studies [33,63,64]. This method takes the principle of minimum factor limitation as the logical starting point, following the principle that the scarcest resources and prospect factors determine the carrying capacity; it emphasizes that a single factor has a decisive impact on carrying capacity [63,65], which is similar to the barrel effect. The limiting factors include food, land, water, and ocean, as well as environments such as water and atmosphere. This method has two application paths. One is to take the limiting factors directly as the analysis basis for regional RECC [42,66,67]; the other is to set the limiting factors as the basis for RECC problem area identification [68,69].

### 4.2. The Multifactor Synthesis Method

Since the 1990s, with technology development and the diversification of resources and environmental supply, it became difficult to precisely reflect the population-carrying status of a country or region using the least limiting factor method [70]. Scholars began to address the impact of multiple factors on the RECC and created the multifactor synthesis RECC evaluation method. This method takes the comprehensive effect principle as the logical starting point, and emphasizes that many subsystems such as socio-economy, resources, environment, and people are interrelated, compensated, and jointly determine the regional RECC [71]. The method includes an RECC definition, conceptual framework design, index selection, and comprehensive assessment [23]. The conceptual framework design is the major content, and it determines the indicator system construction. At present, there are many conceptual frameworks, such as the resource–environment framework [72], resource–environment–socioeconomic–ecological framework [73], territorial space framework [74], or support–pressure–regulatory framework [75]. 

The multifactor synthesis method completes a comprehensive evaluation through the steps of single-factor measurement, weight determination, and synthesis evaluation [76]. The comprehensive evaluation methods include the analytical hierarchy process, principal component analysis, projection pursuit, and so on [77]. The results of the multifactor synthesis method are the spatial distribution characteristics of the dimensionless RECC value.

### 4.3. The Pressure-Carrying State Spatial Method

According to existing research, the impact factors of RECC can be divided into two types: one is natural factors and the other is socio-economic development factors, which are usually regarded as carriers’ bodies and pressure bodies [78]. RECC is therefore, interpreted as a quantitative expression of the pressure imposed on the carrier, and the pressure–carrying state space method of RECC is proposed and gradually developed based on this view [79]. In essence, the pressure–carrying state spatial method enriches the theoretical implications on RECC from the human–land relationships and the supply–demand relationships, based on multiple factors [45,80]. In practical applications, this method calculates RECC by comparing the size of the carrying state and the pressure state [81,82] or assesses RECC using a coupling coordination degree model [83] and comprehensive assessment model (e.g., analytic hierarchy process model, entropy weight TOPSIS model) [23]. Therefore, the RECC results obtained by this method may be dimensional or non-dimensional. 

### 4.4. The Relative Carrying Capacity Evaluation Method

Taking the per capita resource ownership, consumption, or resource stock in the sample area, interior area, or similar area as the reference standard, we can use the relative carrying capacity evaluation method to assess the RECC in a target area [84]. This method takes the relative RECC as the logical starting point and emphasizes the relativity, comparability, and reference of RECC [85]. The evaluation steps are as follows: First, it assumes that the resources and environment in the reference region have been stable and comparable for a long time, and the resources and environment in the target area is difficult to accurately estimate. Second, the per capita resource ownership or consumption in the reference area is calculated as a standard of comparison. Third, the difference between the per capita resource ownership or consumption of the target area to be calculated and compared with the reference area, and the distance between them is used as the RECC value [86,87]. The biggest difficulty of this method lies in how to determine a reliable threshold reference region [88]. The global or national RECC average is often taken as the reference standard, but whether this standard is sustainable needs to be tested by practice [80].

### 4.5. The Ecological Footprint Evaluation Method

In the context of economic globalization, the flow of materials, energy, and other resources among regions has become normal, and countries are gradually moving toward an interconnected unity. In view of this, taking the countries classified as Asian Tigers, with small land areas but rapid economic development as a case study, Rees [89] proposed the ecological footprint RECC evaluation method. This method regards the life cycle of human pressures or burdens on resources and the environment as the logical starting point, and uses land area to represent the degree of human resource consumption and waste discharge to the ecological environment [88,90]. The measurement steps include: (1) determine the ecological footprint parameters of different resource elements, that is, set the per capita consumption, equilibrium factor, yield factor, etc.; (2) measure the ecological footprint; (3) judge the RECC state (surplus or deficit) according to the ecological footprint [91]. The comprehensive calculation path includes process analysis and a calculation framework based on input–output analysis. The former includes a comprehensive method applicable to the top-down calculation at the national level and the bottom-up calculation applicable to provinces, enterprises, households, and individuals as the assessment unit [92]. 

### 4.6. The System Dynamic Method

RECC is a dynamic indicator that changes over time. The prediction of the RECC can help support LUP. By introducing the above evaluation methods to emphasize the system complexity and dynamic predictability of RECC, Zhu [93,94] and Yang [94] introduced a system dynamics (SD) model pioneered by Forrester [95] to simulate the dynamic change of the RECC. Thus, the SD method of RECC was formed. Historical statistical data, empirical parameters (e.g., water consumption per capita, food per capita, construction land area per capita) and monitoring data (e.g., pollutant content in water, soil, and air) are basic data. It is necessary to clarify the relationships between stock, flow, rate variables, constants, feedback loops, and other information for scenario simulation. Available software for this purpose includes Vensim, Anylogic, and Stella [96]. The system dynamics prediction method has been widely used in RECC predictions, such as in atmospheric environmental carrying capacity simulation [97,98], land resources carrying capacity simulation [93], water resources security simulation [61], and water environmental (Su et al., 2019; Hu et al., 2021) or comprehensive RECC simulation [99]. 

In summary, the advantage of static assessment methods is that they can help identify key limiting factors, which are helpful to formulate RECC improvement schemes in LUP [36]. However, the static assessment methods have difficulty in monitoring dynamic trends, providing early warnings, and evaluating cost–benefit relationships under different policy scenarios. The SD method of RECC can help overcome these shortcomings, however, its application requires substantial historical data, which might be unavailable for many cities/regions; the existing studies focus on the impact of socio-economic development and the supply and demand on RECC, and few studies discuss the influence of stakeholder’s actions on RECC.

## 5. Interaction Pathways between RECC and LUP

### 5.1. RECC Evaluation as a Key Tool of LUP

**Figure 4:**
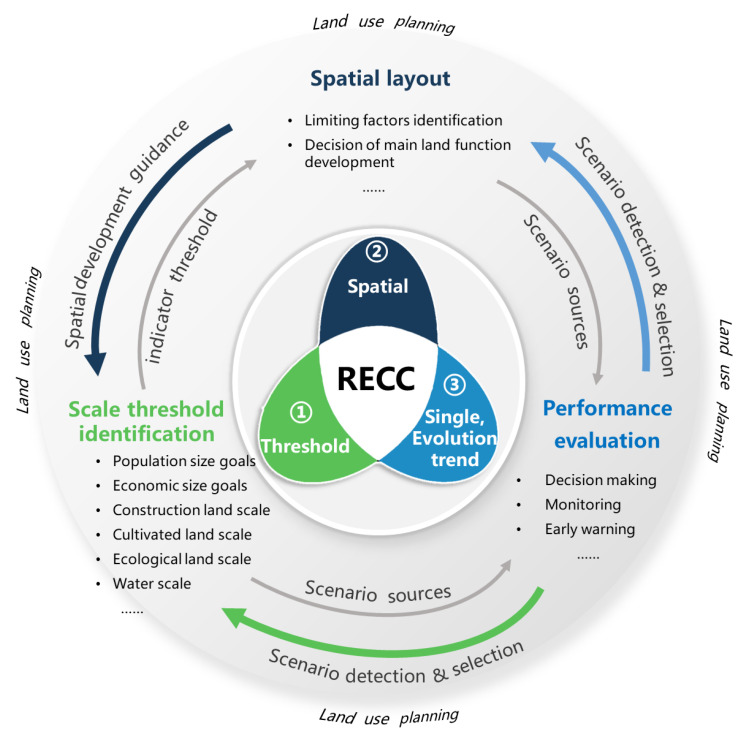
The pathways of RECC assessment as a tool of LUP.

The result of the RECC assessment determines how RECC is applied to LUP. The types of RECC results can be divided into dimensional and non-dimensional according to the unit of RECC. Meanwhile, the types also can be divided into RECC spatial distribution characteristics and total RECC change trends according to the form of expression. According to these result types, at least three pathways of RECC practical applications in LUP can be identified. The contents and interrelations of the three pathways are shown in Figure 4.

*Pathway 1, RECC as a tool of scale threshold identification in LUP.* Scale threshold identification mainly refers to the maximum population size, economic size, or the using of scale thresholds for water, land, ecology, and energy, which can be calculated based on RECC thresholds. This is the main pathway of RECC application in LUP. The key to this path is to set up clear resources and environment use thresholds [2,59]. For example, the goal of maximum population size can be measured by the comprehensive carrying capacity [100]. When the RECC is low or even negative, the economic development goals in the regional LUP are also lowered [81]. For instance, Zhou [101] optimized the agricultural production spatial pattern using the RECC evaluation threshold. Zhu [66] used the critical threshold to estimate the land carrying capacity and provide early warning for LUP by looking for the short board carrying capacity factors. Liao [59] used the carrying capacity threshold value to analyze the coordination between urban growth and RECC. However, as the elements of RECC change frequently, leading to great changes in RECC, the applicability and credibility of the pathway in the scale determination process might be weakened.

*Pathway 2, RECC as a tool of spatial layout in LUP.* Spatial layout can be set based on the RECC evaluation results of different spatial units after considering key limiting RECC factors (identified by obstacle degree model) and dominant RECC factors (identified by the multifactor comparison model). For example, Huang [23] and Zhu [23,66] detected the key limiting factors based on the RECC evaluation, and the space control unit is defined with an optimization strategy based on the key limiting factors. Sun [86] and Huang [86,87] made land-use schemes based on areas with weak carrying capacity, which were identified by the relative carrying capacity method. Ding [102] and Zhang [103] proposed the theory and method of China’s major-function-oriented zoning planning based on RECC. This pathway provides an effective reference for improving sustainable regional development in the LUP. However, as RECC is dimensionless, it is difficult to make cross-sectional comparisons in different regions.

*Pathway 3, RECC as a tool of performance evaluation in LUP.* RECC provides a key tool for quantitative comparison of land-use schemes. Based on SD model simulation, RECC can help predict the future status of RECC. Scenario simulation variables in the SD model correspond to key variables in LUP (e.g., the area of construction land, cultivated land, fishery and aquaculture land, etc.) [60,61,104]. Adjusting these key indicators in LUP and running the SD model will help estimate the costs (e.g., government investment, GDP reduction, farmer income reduction, etc.) and benefits (e.g., RECC improvement) in the planning implementation, and help the cost-effective scheme to be selected [61]. In addition, the simulation can facilitate dynamic monitoring and early warning in the LUP implementation process. For example, Huang [105] used the SD model to simulate the evolution of the atmospheric environmental carrying capacity under different policy scenarios, providing a reference for local governments to formulate atmospheric environmental control policies. Gao [60] simulated the dynamic evolution of land carrying capacity in rural settlements near the Three Gorges reservoir based on the SD model. Hu [106] constructed an integrated assessment system for the water environment carrying capacity based on the SD model. However, this method requires much historical data, and has difficulty in identifying subsystems, establishing a credible relationship, and dealing with complicated interactions, etc.

These three pathways are closely linked and can be complementary. Pathway 1 provides an indicator threshold for Pathway 2, and Pathway 2 provides a spatial develop guidance for Pathway 1. Pathways 1 and 2 simultaneously provide scenario-setting schemes for Pathway 3, while Pathway 3 provides a tool of dynamic monitoring and early warning functions for Pathways 1 and 2.

### 5.2. RECC Improvement Based on LUP

**Figure 5:**
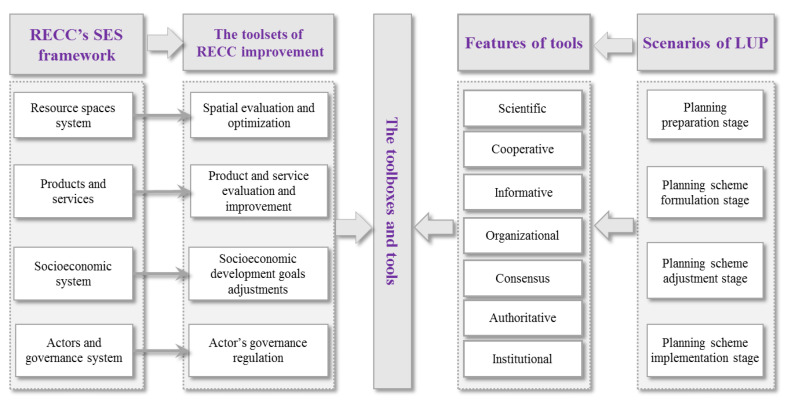
The framework of RECC improvement tools based on LUP.

Based on the SES RECC framework, we introduced the Policy Toolbox research paradigm [107], summarized the main paths of existing LUP tools to improve RECC, and established the multidimensional, multilevel, and multifactor Policy Toolbox for RECC improvement. The toolbox framework is shown in Figure 5, which considers the following five aspects: toolsets, toolboxes, tools, features of tools, and planning application scenarios. RECC is influenced by resource and environment supply, socioeconomic demand, and stakeholder action. RECC is usually improved through adjusting these factors [22,61]. Therefore, the RECC SES framework is regarded as the fundamental basis for toolset construction, which includes the dimensions of the resource spatial system, products and services, the socioeconomic system, actors, and the governance system. Correspondingly, the toolsets of RECC improvement include spatial evaluation and optimization, product and service evaluation and improvement, socioeconomic development demand regulation, and regulation of the actors’ behavior. Combining the elements of the SES framework with the LUP tools, we have established several toolboxes for improving RECC. Table 2 shows the types of the RECC improvement tools based on LUP. While evaluating RECC, once the carrying capacity is found to be overloaded, we can use these tools to optimize the supply and demand factors concerning the RECC. However, the improvement of RECC might be at the cost of socioeconomic development. Therefore, it is necessary to compare the costs and benefits of the scenario of each policy tool, so as to select the most suitable tools.

In the Policy Toolbox, the tools can be classified as scientific, cooperative, informative, organizational, consensus, authoritative, institutional, etc. Scientific tools refer to those characterized by technical and professional requirements in related fields. Cooperative tools denote the top-down or bottom-up cooperation between stakeholders. Informative tools are those supported by advanced information technology. Organizational tools mean those in which stakeholders work together to accomplish a task. Consensus tools indicate those using media to disseminate information to achieve consensus. Authoritative tools reflect publicity, responsibility, and service based on state power. Institutional tools emphasize the characteristics of compliance with laws or rules (e.g., regulations, judicial interpretations, departmental and local regulations, and other normative documents). 

## 6. Summary and Discussion

This article firstly reviewed the research history RECC and its concept and methods evolution, which can provide reference for further improving the theory and method system of RECC. Secondly, based on the SES framework, a new framework is novelty proposed to explain the formation mechanism of RECC combining the governance considerations, which has made contributions to enrich the research perspective of RECC and its theoretical system. Further, the interaction path between RECC and LUP is constructed according to the new RECC framework and a policy toolbox for improving RECC, which will provide a comprehensive and systematic practical application path for improving RECC and promoting regional socioeconomic-ecological coordinated sustainable development.

In sum, the concept of carrying capacity has been developed from the traditional single carrying capacities of water, grassland, or cultivated land, to the comprehensive carrying capacities of resources, environment, and ecology. Meanwhile, the RECC quantitative evaluation and measurement system presents a diversified development trend. In addition, a mutual interaction system has been formed between RECC and LUP. However, we found the following four deficiencies in the current research: 

(1) The theoretical basis of RECC research lacks a unified comprehensive framework. In existing studies, the scientific basis for the resource carrying capacity is the sustainable yield and the limitation of production and living scale, and the scientific basis for the environmental carrying capacity is the environmental supporting capacity, clean production, and lifestyle. These two are independent of one another, and a basic theoretical framework applicable to the unified RECC assessment has not been formed. 

(2) The role of stakeholder in the evolution of RECC has not been sufficiently addressed. The actions of the government, the market, the collective, the public, and other stakeholders have significant impacts on the supply and demand of RECC. However, at present, few studies have taken governance actions as a key element and added them into the theory and method in the RECC assessment and dynamic simulation. As a result, it is difficult to evaluate the real impacts of government, market, and public behaviors on carrying capacity and to provide policymaking decision support.

(3) The research standards of evaluation object, range, and threshold are not the same in quantitative evaluation methods. Due to differences in these standards, the quantitative carrying capacity evaluation results are often highly uncertain, especially for dimensional and dimensionless results, which make the evaluation results incomparable between regions. In addition, research on open, dynamic, and systematic quantitative evaluation and measurement methods are relatively weak. Most of the existing studies focus on assessment and measurement methods of static and closed system carrying capacities. Although some scholars have paid attention to dynamic assessment and measurement methods based on system dynamics, the existing studies are far from sufficient.

(4) A systematic interaction system has not been established between RECC and LUP. RECC is a quantitative index to measure the social and ecological sustainable development of a region, and LUP is a key tool of regional social and economic development and ecological protection. RECC evaluation provides scientific support for LUP, while the latter can provide many tools of contributing to RECC improvement. However, research focusing on systematically explaining the interaction between LUP and RECC has been insufficient [1,2,45,119].

## 7. Conclusions

The evolution and development of RECC not only reflects our deepening understanding of the relationship between humans and nature, but also expresses the development of our understanding and response to resource and environmental limitations under different development stages and constraints [20]. Carrying capacity has increasingly become a critical concept and a key indicator to describe the limits of development, as well as a constraint condition for the size of regional populations and economies. Given the defects of the current research of RECC and LUP, further research needs to be conducted in the following areas: 

(1) In-depth study of the theoretical framework for a unified comprehensive RECC. A solid theoretical framework can better guide the selection of RECC research methods and practical application pathways. This work primarily constructs a RECC theoretical framework using SES and attempts to deconstruct the interactions between multiple subsystems such as resources and environment, socioeconomic development, stakeholder actions, etc., which can be further studied in the future. Further, how to further analyze the micro mechanism between the internal elements of each subsystem should be given enough attention.

(2) More attention should be paid to the role of stakeholder actions in RECC assessment, measurement, and improvement. The collaboration of stakeholders is critical for good governance. Meanwhile, it is necessary to deeply explore the ways, degrees, and benefits of stakeholders’ influence on RECC by SD model simulation. However, in the research process, how to find latent or substitute variables of stakeholder actions and collect enough data to support model operation will be the biggest challenge of this research direction.

(3) More research on dynamic, and systematic measurement and prediction methods should be conducted. Standardized evaluation and measurement methods are the basis for cross-regional and cross-border comparability of research results, while open, dynamic, and systematic measurement and prediction methods are the basis for monitoring, early warning, and intelligent control. At the same time, more attention should be paid to the unreliability of dynamic prediction results caused by random changes of basic elements.

(4) A systematic interactive application system between RECC and LUP can be established. RECC results can provide reference for better LUP. Similarly, LUP should set up the goal of RECC improvement to promote sustainable development. In addition, attention should be paid to the simplicity and practicability tools of the application path of “RECC improvement based on LUP”.

This review article has revealed that the reports of analytical results can be highly inconsistent between publications. At present, there are many examples of research on RECC comprehensive evaluation. However, most studies have not reported their application methodology, leading to an unclear interaction pathway between RECC and LUP. This may be due to insufficient literature reading in our work. Overall, RECC is a socioeconomic and ecological sustainable development issue that requires more attention, not only from international organizations and national governments, but also from academic circles. This study proposes a combined RECC and SES framework and the interaction pathway framework between RECC and LUP which can be used and tested in future research work. 

## Figures and Tables

**Table 1:** Evolution of the RECC concept.

Background	Concept	Connotation
Human ecology	Carrying capacity	The limit on the number of individual organisms under a specific environmental condition (mainly referring to the combination of living space, nutrients, sunlight, and other ecological factors) [26].
Grassland degradation	Grassland carrying capacity	The maximum number of animals that can be carried within a pasture [27].
Ecological security	Biomass carrying capacity	The capacity of an ecosystem to carry the maximum amount of a particular biomass at a time [28].
Deal with disaster like hunger, war, poverty	Human carrying capacity	The maximum population that a city or urban agglomeration can carry under certain resource and environmental constraints on the premise of meeting human’s ever-increasing needs for a better life [30].
Population soaring and land resource scarcity	Land resources carrying capacity	The productive capacity and the maximum population that can be carried by regional land resources [32,48].
Sustainable development of resources	Resources carrying capacity	The capacity of resources to carry the basic survival and development of the population in a region [49].
Water shortage induced by drought or pollution	Water resources carrying capacity	The maximum population and the intensity of industrial and agricultural production activities that can be carried by regional water resources [50].
Mineral resource shortage	Mineral resources carrying capacity	The maximum population and aggregate economy that can be carried by the stock of mineral resources in the foreseeable period, under the conditions of science and technology [39].
Serious environmental pollution	Environmental carrying capacity	The self-purification capacity of water, atmosphere, and soil environments to carry the pollutant discharge capacity of human life and economic development [35,36,51].
Serious ecological damage	Ecological carrying capacity	The capacity of an ecosystem to carry the maximum human socioeconomic activities [40].
Post-disaster reconstruction planning Territorial spatial planning	Resources and environmental carrying capacity	The capacity of resources and environment (including water, soil, and ecology) to carry the maximum human socioeconomic activities [41,42].
Coordinated development of product–living–ecological spaces	Product–living–ecological carrying capacity	A capacity complex composed of land resources and ecological environment to carry the economic activities for a certain standard of living [44].
Rural revitalization	Resources and environmental carrying capacity of rural and township development	The supporting capacity of the rural and township carrier (including the resources and environment of water, soil, ecological) [22,45].
Cultural protection	Culture carrying capacity	The maximum scale, intensity, and speed of human social activities that the cultural system can carry under the premise of maintaining coordinated and sustainable development of people and nature within a certain period and region [43].
Regional and urban sustainable development	Economic carrying capacity	The economic activity capacity that a city or urban agglomeration can carry under certain resource and environmental constraints on the premise of ensuring high-quality economic development [24].
	Traffic carrying capacity	The supporting urban road car carrying capacity; overload is congestion [46].
Tourism sustainable development	Tourism carrying capacity	The maximum number of tourists that a tourist destination system can carry in a certain period of time without harmful changes [47].

**Table 2:** RECC improvement tools in LUP.

Toolsets	Toolbox	Tools	Features of Tools and Scenarios of LUP	References
*Spatial evaluation and optimization toolsets*	Single-type spatial constraint evaluation and optimization (SCEO) toolbox	Water SCEO tools; Forestland SCEO tools; Grassland SCEO tools; Cultivated land SCEO tools; Construction land SCEO tools. ……	*Features:* scientific, informative *Planning preparation stage:* The comprehensive assessment method identifies the status quo of all spaces. *Planning scheme-making stage:* Determine the scale bottom line (upper limit, lower limit) of space development and protection using the carrying capacity, delimit the scale space of the bottom line according to the spatial quality evaluation, and identify the protection and development red line.	[1,57,58,74,104]
	Comprehensive spatial constraint evaluation and optimization toolbox	Agriculture space SCEO tools; Ecological space SCEO tools; Production space SCEO tools; Living space SCEO tools. ……		[42,108,109,110]
*Product and service evaluation and improvement toolsets*	Product evaluation and promotion toolbox	The evaluation and promotion tools of gross domestic product (GDP) output, arable land output, fishery output, livestock output, forestry output……	*Features:* scientific, informative *Preplanning stage:* Obtain the spatial distribution characteristics or long-term evolution of resources and environmental products and services in the region through the method of sample point measurement. *Planning scheme formulation stage:* analyze and identify the key restrictive factors and regions that affect the comprehensive carrying capacity, and formulate plans to improve the status of products and services from the aspects of improved variety, scientific and technological inputs, source control, and comprehensive treatment and restoration.	[23,90,99]
	Service evaluation and improvement toolbox	Water environment assessment and improvement tools (COD, BOD, NH_3_–N, TP, TN, etc.); Improved assessment of atmospheric environment (PM2.5, CO_2_ emissions, etc.); Soil environment assessment and improvement tools (heavy metal pollution, pesticide and fertilizer use exceed the standards); Ecosystem service evaluation and promotion tools (windbreak and sand fixation, soil conservation, carbon sequestration and oxygen release, biodiversity, etc.). ……		[42,50,69,72,75,97,98,105,111,112]
*Socio-economic development demand toolsets*	Economic development toolbox	The goals of per capita GDP adjustment analysis; The goals of industry positioning and adjustment analysis. ……	*Features:* Authoritative, organizational *Preplanning stage:* preliminary formulation of economic and population development targets based on historical data trends *Planning scheme adjustment stage:* Coordinate the proposed economic and population development goals according to the RECC calculation results.	[24,76,81,113]
	Population toolbox	The goals of total population size adjustment analysis; The goals of urban population adjustment analysis. ……		[22,68,81,100,114]
*Actors’ governance toolsets*	Government: Institutional toolbox	National strategic positioning (ecological protection, food security, rural revitalization, economic development, coordinated development of production-living-ecology etc.); Land development rights (Urban, agricultural, and ecological space; protection and development zone and scale demarcations; floor area ratio control; policy of balance between occupation and subsidy); Water rights tools (water rights trading system, river basin horizontal ecological compensation system). ……	*Features:* Institutional, authoritative, cooperative *Preplanning stage:* Determine planning orientation, basic methods *Planning scheme formulation stage:* identify the main content and direction of the planning	[20,44,60,80,88,115]
	Government: Resources exploitation toolbox	Fallow field policy; Grazing prohibition policy; Fishing ban policy; Logging prohibition policy. ……	*Features:* Institutional, authoritative, cooperative *Planning scheme implementation stage:* Implements access and development intensity limitation measures, improves the self-organizing restoration ability of resources and environmental products and services in the target region.	[2,42,47,69,76,106]
	Government: Pollution discharge toolbox	Industrial and agricultural production pollution emissions; Urban and rural domestic sewage discharges; ……	*Characteristics:* Institutional, authoritative, cooperative (top-down) *Planning scheme formulation stage:* Based on existing conditions, formulate production and domestic emission standards *Planning scheme implementation stage:* Encourage green production and life by limiting pollution discharges, and identify producers and lifestyles with serious pollution discharges, to improve environmental demand.	[50,58,61,69,98,115,116,117]
	Government: space integrated development toolbox	Scientific and technological means (scientific and technological investment and popularization of science and technology); Infrastructure investment; High-quality farmland development; Low-efficiency industrial land consolidation; Homestead retreats; Mine restoration; Landslide and mud–rock flow regulation; Stagnant water restoration; Farmland to forest conversion; River basin horizontal ecological compensation;……	*Features:* institutional, authoritative, cooperative (top-down) *Planning scheme stage:* use the key constraint tools to develop different planning schemes. *Planning scheme implementation stage:* According to the key constraints on the overloaded areas, tools are adopted to improve the resource supply capacity and environment products and services.	[23,45,58,98,99,117,118]
	Market action toolbox	Increasing investment in green and ecological industries; Increasing local technology innovation jobs; Increasing green technology research and development jobs; ……	*Features:* organization, consensus, cooperation (bottom-up) *Planning scheme formulation and adjustment stage:* the market, the collective, and the public can participate in the scheme formulation and adjustment process through publicity, soliciting opinions, holding press conferences, and other forms. *Planning scheme implementation stage:* The market, the collective, and the public are the core participants in planning implementation, and their actions will be the key to whether the planning can be implemented to improve the carrying capacity.	[23,47,55,65,114,117]
	Collective organizational capacity improvement toolbox	Publicize information; Organize technical training and collective meetings; Develop outreach capacity; Cultivate professionals; ……		
	Public participation toolbox	Participate in technical training; Self-organizing collaborative cooperation; Increased awareness of resource and environmental protection; Mutual supervision; ……		

## Data Availability

Data sharing not applicable. No new data were created or analyzed in this study. Data sharing is not applicable to this article.
